# Loss of ATRX in Chondrocytes Has Minimal Effects on Skeletal Development

**DOI:** 10.1371/journal.pone.0007106

**Published:** 2009-09-23

**Authors:** Lauren A. Solomon, Jennifer R. Li, Nathalie G. Bérubé, Frank Beier

**Affiliations:** 1 Department of Biochemistry, University of Western Ontario, London, Ontario, Canada; 2 Children's Health Research Institute, Victoria Research Laboratories, London, Ontario, Canada; 3 Skeletal Biology Group, University of Western Ontario, London, Ontario, Canada; 4 Department of Paediatrics, University of Western Ontario, London, Ontario, Canada; 5 Department of Physiology and Pharmacology, University of Western Ontario, London, Ontario, Canada; University of Michigan, United States of America

## Abstract

**Background:**

Mutations in the human *ATRX* gene cause developmental defects, including skeletal deformities and dwarfism. *ATRX* encodes a chromatin remodeling protein, however the role of ATRX in skeletal development is currently unknown.

**Methodology/Principal Findings:**

We induced Atrx deletion in mouse cartilage using the Cre-loxP system, with Cre expression driven by the collagen II (Col2a1) promoter. Growth rate, body size and weight, and long bone length did not differ in Atrx^Col2cre^ mice compared to control littermates. Histological analyses of the growth plate did not reveal any differences between control and mutant mice. Expression patterns of Sox9, a transcription factor required for cartilage morphogenesis, and p57, a marker of cell cycle arrest and hypertrophic chondrocyte differentiation, was unaffected. However, loss of ATRX in cartilage led to a delay in the ossification of the hips in some mice. We also observed hindlimb polydactily in one out of 61 mutants.

**Conclusions/Significance:**

These findings indicate that ATRX is not directly required for development or growth of cartilage in the mouse, suggesting that the short stature in ATR-X patients is caused by defects in cartilage-extrinsic mechanisms.

## Introduction

ATR-X syndrome (Alpha-Thalassemia/Mental Retardation, X-linked) is a human disorder caused by mutations in the *ATRX* gene[1], [2]. Clinical manifestations include severe psychomotor and mental retardation, characteristic facial features, urogenital abnormalities, skeletal deformities and α-thalassemia [2]. Over 200 male ATR-X syndrome cases have been reported [3], and female carriers are unaffected due to the skewed pattern of X-inactivation [4]. ATR-X syndrome patients display a wide range of skeletal abnormalities, and 66% of patients show dwarfism [4]. About half of ATR-X patients have spinal deformities such as kyphosis or scoliosis [2]. Delayed bone age is characteristic of most cases studied by thorough radiological investigation [2]. For some patients, these skeletal abnormalities are apparent at birth, for others they manifest later in life, during the pubertal growth spurt [4]. Other skeletal deformities commonly seen in ATR-X patients include clinodactyly, brachydactyly, tapering of the fingers, overlapping digits, and 40% of patients have foot deformities [2]. A single case of bifid thumb has been reported [4]. Despite a broad characterization of the variety of physical and mental phenotypes, very few genotype/phenotype correlations have been established, and none are associated with the severity of skeletal deformities [5]. The molecular and genetic basis of these phenotypes are therefore unknown and it is unclear if these defects are due to a direct role of ATRX in the skeleton. Global knockout of *Atrx* using a ubiquitous GATA-Cre system leads to placental defects and embryonic lethality [6]. Conditional ablation of ATRX in the forebrain causes p53-dependent apoptotic cell death during embryogenesis, resulting in a smaller brain at birth [7], [8], while ATRX deficiency in the retina induces specific loss of interneurons [9].

The ATRX protein contains a domain displaying high homology to SNF2 (Sucrose Non-Fermenting 2) proteins, suggesting a role as a chromatin remodeling protein [10]. The SNF family of proteins is involved in transcriptional regulation, maintenance of chromosome stability during mitosis and processing of DNA damage [11]. ATR-X patient mutations are generally hypomorphic and do not cause chromosomal instability[4]. However, depletion of ATRX in mammalian cells leads to defects in chromosome cohesion and mitotic progression [12]. In addition to its role during mitosis, ATRX appears to be involved in the regulation of gene expression [13].

The development of long bones occurs through endochondral ossification, a highly regulated multi-step process initiated when pluripotent mesenchymal cells aggregate to form the beginning of a cartilage model [14]. Under the control of several transcription factors, such as Sox9, Sox5 and Sox6 [15], [16], mesenchymal cells differentiate into chondroblasts, which produce large quantities of type II collagen (Col2) [17]. Chondroblasts mature to form chondrocytes, which undergo rapid proliferation along the longitudinal axis of the future bone, forming the cartilage growth plate [18]. The differentiated chondrocytes in the center of the cartilage model undergo hypertrophy, increasing in cell size and secreting type X collagen (ColX) [19]. The hypertrophic chondrocytes encased in calcified ECM secrete vascular endothelial growth factor (VEGF), which recruits blood vessels bringing osteoblasts and osteoclasts to form the primary ossification center; and eventually undergo apoptosis [19]. Chondrocytes on either end of this primary ossification center continue to proliferate, enter hypertrophy and undergo apoptosis, thus allowing the bone to grow longitudinally. Finally, woven bone is laid down in the area of apoptotic chondrocytes and is remodeled by osteoblasts and osteoclasts to form lamellar bone [20].

To examine whether skeletal defects in ATR-X patients could be due to a requirement for ATRX in cartilage development, we examined the outcome of cartilage-specific inactivation of ATRX in mice, using the Cre-loxP system. Our findings demonstrate that loss of ATRX specifically in chondrocytes induces minor skeletal defects, but does not affect growth plate morphology and bone growth.

## Results

### ATRX is expressed in chondrocytes

**Figure 1:**
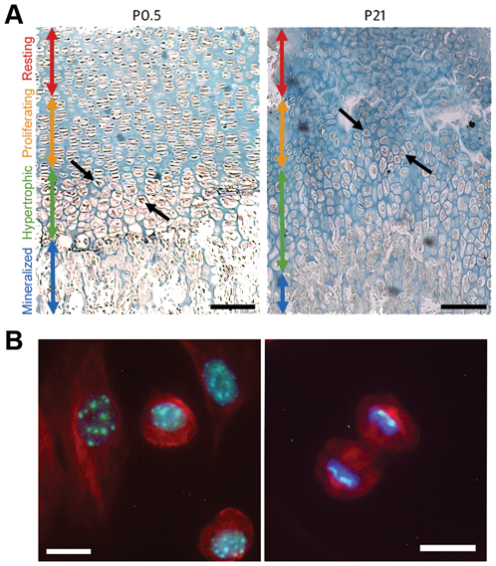
Expression and localization of ATRX in growth plate chondrocytes. (A) Immunostaining of ATRX in humerus growth plates of P0.5 and P21 mice. ATRX is seen in the nuclei of resting, proliferating and early hypertrophic cells in the growth plates (arrows). Scale bar: 100 µm. Regions of the growth plate are identified as resting (red arrow), proliferating (yellow), hypertrophic (green) and mineralized (blue). (B) Immunofluorescence detection of ATRX in primary mouse chondrocytes isolated from E15.5 long bones. Merged image of ATRX (green), DAPI (blue) and alpha-tubulin (red) reveals a punctate ATRX staining pattern restricted to the nucleus during interphase (left panel) and specific localization to condensed chromatin during mitosis (right panel). Scale bar: 10 µm.

**Figure 2:**
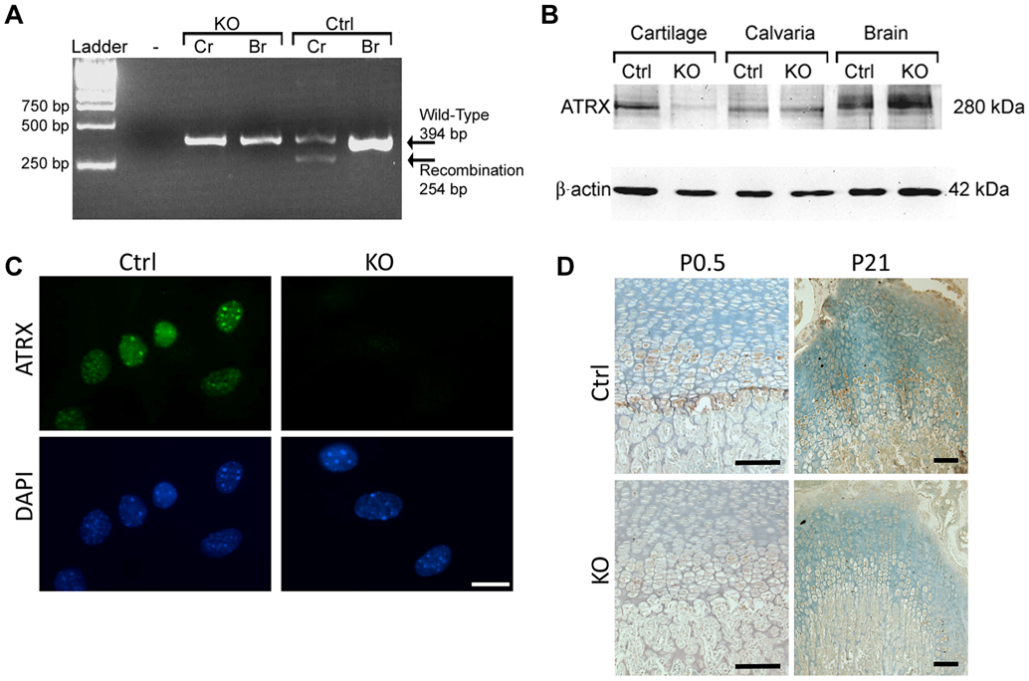
Levels of ATRX are decreased in the cartilage of *Atrx^Col2Cre^* mice. (A) RT-PCR analysis of RNA isolated from newborn rib cartilage (Cr) or brain (Br) from *Atrx^Col2Cre^* males (KO) and control littermates (Ctrl). Amplification was performed with primers flanking the loxP sites, in introns 17 and 20. The expected amplicon of 394 bp was obtained in control and an additional amplicon of 254 bp was obtained in KO cartilage caused by the recombination event. The levels of the recombined product are low, as described previously [6], [7], suggesting that this product is unstable. (B) Immunoblotting for Atrx from proteins isolated from newborn control and KO rib chondrocytes, calvariae and brain tissue. Atrx expression is greatly reduced in KO chondrocytes, but unaffected in calvariae and brain of KO animals. Beta-actin was used as a loading control. (C) Immunofluorescence detection of ATRX in control and *Atrx^Col2Cre^* primary chondrocytes isolated from newborn long bones shows the loss of ATRX protein in KO chondrocytes. Scale bar: 10 µm. (D) Immunohistochemistry on growth plates from P0.5 and P21 mice. ATRX staining is reduced in KO growth plates. Scale bar: 100 µm.

Expression of ATRX in chondrocytes was assessed using *in vitro* and i*n vivo* models. Immunohistochemistry of newborn (P0.5) and three week-old wild type mice demonstrated that ATRX is expressed throughout the cartilage growth plate. Strong nuclear staining of ATRX was apparent in all cartilage cells, but was most prominent in early hypertrophic chondrocytes. Lower levels of staining were observed in terminally differentiated cells (Figure 1A). Primary chondrocytes in monolayer culture were stained for ATRX by immunofluorescence. In interphase, we observed a punctate staining pattern within the nucleus that corresponded to bodies stained intensely with DAPI (Figure 1B, left panel). Alpha-tubulin (red) was used to visualize the location of the cytoplasm and to identify microtubules during cell division. During metaphase, ATRX was present exclusively at the edges of the aligned chromosomes, similar to the localization at pericentromeric heterochromatin reported in other cell types (Figure 1B, right panel) [12], [21]. Western blots of primary cultured chondrocytes showed that full-length ATRX was expressed in cartilage (Figure 2B).

### Loss of ATRX in mouse chondrocytes does not affect viability or growth

We utilized the Cre-LoxP system to generate mice with cartilage-specific inactivation of the *Atrx* gene. Female mice previously engineered with loxP sites flanking exon 18 of *Atrx* [7] were mated with male mice expressing Cre recombinase under the control of the mouse collagen II (*Col2a1*) promoter [22]. Reverse transcriptase PCR (RT-PCR) analysis of ATRX transcripts in cultured primary chondrocytes confirmed a strong reduction in wild type *Atrx* mRNA and the presence of low levels of a shorter transcript resulting from the recombination event in mutant cartilage (Figure 2A). These results confirm previous reports that the mRNA species generated by the recombination event is unstable [6], [7]. Levels of ATRX protein in chondrocytes from ribs of newborn mice were assessed by immunoblotting. ATRX protein amounts were substantially decreased in *Atrx^Col2cre^* chondrocytes (Figure 2B). In contrast, ATRX protein levels in brain and bone (calvariae) were unchanged in mutant mice, validating the tissue specific activity of the *Col2a1* Cre (Figure 2B). Loss of ATRX protein was further confirmed by immunofluorescence of cultured chondrocytes isolated from *Atrx^Col2Cre^* and control littermate mice (Figure 2C) and by immunohistochemistry of *Atrx^Col2Cre^* and control growth plates (Figure 2D). These data showed that our breeding scheme resulted in specific and efficient loss of ATRX protein in cartilage.

**Figure 3:**
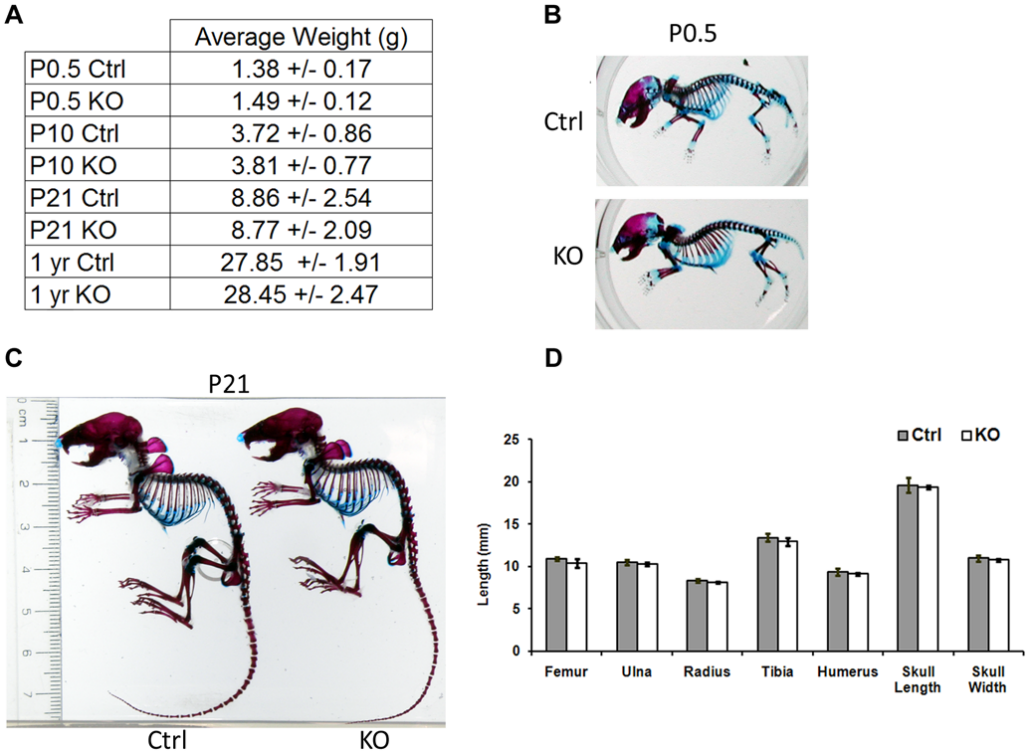
Effects of ATRX loss-of-function on growth and development of the skeleton. (A) Weight measurements from control and KO mice. No differences were seen in the growth of *Atrx^Col2Cre^* mice compared to control littermates, and weight gain was normal at all stages of development. (B, C) Skeletal stains of newborn and p21 control and KO mice showing cartilage (blue) and bone (red) proportions. Extent of limb ossification and skeletal proportions between control and mutant pups were unaffected at birth and at three weeks (D) Long bone lengths and skull proportions were measured from four control/KO littermate pairs at p21, and found to be unaffected by ATRX loss.

*Atrx^Col2Cre^* mice were obtained at expected Mendelian ratios, indicating that loss of ATRX in cartilage does not result in embryonic or neonatal lethality (59 control males, 61 KO males out of 29 litters). In addition, *Atrx^Col2Cre^* mice showed no significant difference in growth when weighed daily from birth to weaning and again at one year of age (Figure 3A), or in body length at three weeks of age compared to wild-type littermates (Ctrl: 6.30 cm STD: 0.21, KO: 6.17 cm STD: 0.38, p>0.05). Body length at 6 and 12 months was also unaffected (data not shown). Null males were capable of mating and producing offspring when backcrossed to floxed females to produce second-generation tissue-specific knockout mice.

Skeletal preparations from newborn and weanling (21 day old) mice showed no change in skeletal morphology, bone length or extent of ossification, as determined by the ratio of Alizarin red to Alcian blue staining in the long bones (Figure 3B,C). Length of tibia, femur, radius, ulna, and humerus were measured, as well as the length and width of the skulls. Average measurements obtained from four independent littermate pairs revealed no significant changes in the length of long bone elements between control and mutant mice (Figure 3D).

### Growth plate morphology is not affected by the loss of ATRX

**Figure 4:**
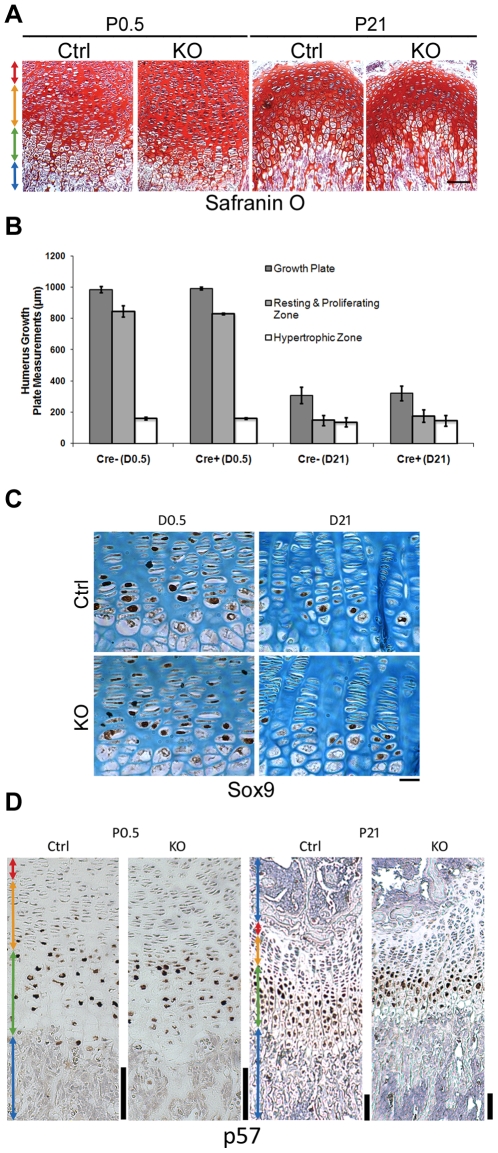
ATRX-null growth plates are indistinguishable from controls. (A) Growth plate sections from control and *Atrx^Col2Cre^* mice at P0.5 and P21 were stained with safranin-O/Fast green, demonstrating unaltered growth plate architecture and morphology in mutant mice. Scale bar: 100 µm. (B) No significant difference in the length of the resting, proliferating or hypertrophic zones could be detected between genotypes at birth or weaning (N = 3 littermate pairs; two-tailed T-test). Error bars depict standard error of the mean (SEM). (C) Expression of Sox9, an early chondrocyte differentiation marker expressed in resting and proliferating chondrocytes, is not altered in the absence of ATRX at P0.5 or P21. Scale bar: 50 µm. (D) Immunohistochemistry for the differentiation marker p57 reveals that ATRX loss has no effect on the proportion of chondrocytes reaching terminal differentiation. Scale bar: 100 µm.

Histochemical staining was conducted on paraffin sections of the growth plates of *Atrx^Col2Cre^* and control mice to examine proportions of resting, proliferative and hypertrophic zones. No difference in growth plate architecture or chondrocyte morphology was detected between genotypes at P0.5 or P21 (Figure 4A). In agreement with these data, no significant differences were seen in the length of any growth plate zone in three independent litters (Figure 4B). The early chondrogenic marker Sox9 was expressed in resting and proliferating chondrocytes of control and KO sections, demonstrating that loss of ATRX in growth plates had no effect on Sox9 expression (Figure 4C). To confirm that differentiation was unaffected in mutant chondrocytes, long bone sections were stained for p57, a cyclin-dependent protein kinase inhibitor that is expressed by prehypertrophic, postmitotic chondrocytes [23]. The pattern of p57-positive cells in the mutant growth plates at P0.5 and P21 (Figure 4D) was not different between control and mutant mice, suggesting that loss of ATRX in the cartilage growth plate does not affect terminal differentiation of chondrocytes. In addition, the expression of other cartilage markers such as ColX, Col2, or aggrecan was not altered at P0.5 or at P21 (data not shown), indicating that chondrocyte differentiation was unaffected by loss of ATRX expression in cartilage.

### Conditional loss of ATRX in the mouse skeleton causes minor ossification defects

**Figure 5:**
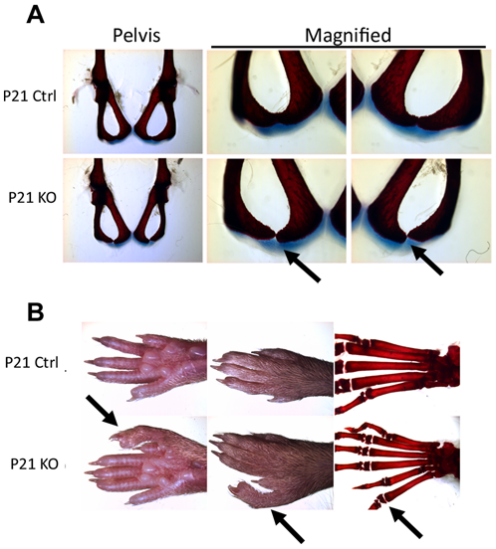
Minor defects observed in *Atrx^Col2Cre^* mice. (A) Pelvis phenotype of P21 control and *Atrx^Col2Cre^* mice. Arrows indicate the site of delayed ossification in mutant mice. (B) Hind limb of mutant mouse with one additional digit, before and after skeletal preparation to stain for cartilage (blue) and bone (red). The additional digit is indicated by an arrow.

Although no overall changes in skeletal size or proportions were seen in the *Atrx^Col2Cre^* mice, minor delays in development were seen in some mice. Half (2/4) of the mutants examined by skeletal staining at day 21 displayed a delay in ossification of the union between the pubis and the ischium (Figure 5A). In those mutant mice where ossification was complete by day 21, the site of union was uneven with a spur-like bone projection. Both phenotypes suggest a mild defect in the ossification of the hip in the *Atrx^Col2Cre^* mice.

A second phenotype observed in one of the 61 *Atrx^Col2Cre^*males was an extra digit in a hind foot (Figure 5B). This digit resembled an additional digit, similar to the fourth digit, rather than a bifurcation of the fifth toe. The toe appeared normal, and in skeletal preparations showed no gross differences from other digits.

## Discussion

In this study, we show that loss of the ATRX protein specifically in cartilage of mice does not cause a major defect in skeletal growth or development. Although ATRX loss in the cartilage of mice was confirmed by immunohistochemistry, immunofluorescence and western blot analyses, we show that long bones from *Atrx^Col2Cre^* mice do not differ from controls in length or growth-plate morphology, and that these mice achieve the same adult length and weight as control littermates. Furthermore, mutant mice are viable and breed normally, with no defects seen in second-generation knockouts.

Whole skeletal stains for ossified bone and cartilage were used to characterize the entire skeleton of *Atrx^Col2Cre^* mice. Qualitative and quantitative study of the control and *Atrx^Col2Cre^* skeletons showed no overall changes in cartilage proportions and lengths of individual elements.

The minor defects observed in our mouse model suggest a mild effect from ATRX deficiency that may manifest only occasionally. This is not unexpected, as the limb and digit phenotypes observed in ATR-X syndrome patients are also highly variable. In a study of sixty-five unrelated patients, one case of bifid thumb was observed [5]. The varied hand and foot deformities in some ATR-X patients suggest that the effect on limb development may differ between individuals, even those with identical mutations [5]. Thorough radiologic investigations of ATR-X patients have shown that the most common phenotypic abnormalities were delayed bone age and coxa valga [24]. The delay in hip ossification seen in our mice may be due to a similar delay in bone age.

Previous studies have shown that loss of ATRX in other tissues in the mouse causes severe and fatal defects [6], [7], [9]. Loss of ATRX in the mouse forebrain has been shown to result in widespread hypocellularity in the hippocampus and neocortex, as well as an overall reduction in forebrain size. These mice exhibit reduced weight and perinatal lethality, demonstrating the importance of ATRX in the brain [7]. Similarly, loss of ATRX in the 16-cell morula stage has revealed a specific and essential role for ATRX in the formation of the extraembryonic trophoblast in mice. Mutant mice showed a dramatic reduction in mitotic cells and early embryonic death at E9.5[6]. A third model examining the role of ATRX in the developing retina showed that ATRX loss-of-function leads to loss of interneurons, specifically, amacrine and horizontal cells. These mice demonstrated a defect in interneuron differentiation and survival, which is associated with functional deficits that may be similar to the subset of ATR-X patients with visual anomalies [9].

This is the first study examining the specific loss of ATRX in the skeletal system, and surprisingly we observed only minor abnormalities. Since these effects are much less severe than in all other cell types examined, despite marked expression of ATRX in wild type chondrocytes, our data suggest cell type-specific requirement for ATRX function in cell survival and differentiation.

One assumption in our model is that destabilization and subsequent breakdown of the *Atrx* mRNA is an accurate model of the hypomorphic gene expression seen in ATRX patients. While our model deletes only the long isoform of ATRX and retains the short isoform (ATRXt), it is unlikely that ATRXt has equivalent functions to the full length protein, as it lacks the functional SWI/SNF domain [25]. In addition, the same floxed *Atrx* mouse line has been used by us and others to successfully conduct loss of function studies in other organs, such as the brain [7], [8] or eye [9]. Since loss of ATRX does not affect cartilage development in mice, it seems likely that the skeletal defects in ATR-X patients are not due to direct effects of the mutant proteins in chondrocytes.

We have used the cartilage-specific collagen II promoter, which is activated around embryonic day 9, for inactivation of the *Atrx* gene. However, there remains a possibility that some of the limb phenotypes seen in ATR-X patients (e.g. patterning defects affecting the digits) are due to an earlier effect, such as a function in formation or outgrowth of the limb bud or in formation of the initial mesenchymal cell template. Since loss of ATRX occurs later in development in our model, we cannot rule out such an earlier function for the protein. Use of an early limb bud-specific promoter, such as the Prx1 limb enhancer [26], to direct *Atrx* inactivation could answer this outstanding question. Additionally, a later deficiency may also contribute to the observed patient skeletal abnormalities. For example, defects in osteoblasts could contribute to delayed bone age seen in patients or reduced overall growth. *Atrx* deletion specifically in osteoblasts could be induced by using the a1(I)-collagen promoter to drive cre expression [27]. These experiments have been initiated in our laboratory.

An alternative explanation for the surprising lack of skeletal defects in our mice is that the skeletal defects observed in patients might be secondary to abnormalities in other organs, such as neuroendocrine defects stemming from ATRX dysfunction in the nervous system [28]. There is much new evidence demonstrating that bone growth can be regulated centrally via cytokines, hormones and transcription factors, including the hormone leptin [29]. Experiments using leptin directly on bone cells have shown no effect on bone remodeling, however intracerebroventricular leptin infusion into leptin-deficient mice leads to an effect on bone mass via the nervous system [29]. Similarly, hematopoietic systems within the bone marrow have been shown to be influenced by the neurohormone melanin and catecholamines [30]. Both factors also have hematopoietic roles, and are present in substantial amounts in the bone marrow, supporting the idea that neural and neuroendocrine factors have a direct effect on the bone microenvironment. Together, these studies suggest that cell defects in the hypothalamus (in the case of leptin) or the pineal gland (in the case of melatonin) may have a central influence on the development and homeostasis of the skeleton.

Importantly, it appears likely that the large variety of skeletal and growth abnormalities in ATRX patients does not have one common cellular origin; instead it appears that some defects (e.g. digit malformations) could be due to alterations in the early patterning of the limb, others (such as delayed bone age) could be caused by defects in osteoblasts (or possibly osteoclasts) and a third group (such as growth retardation) could be caused by abnormal neuroendocrine signaling. These possibilities will require examination as alternative mechanisms for the pathogenesis of ATR-X syndrome.

## Materials and Methods

### Histology and immunohistochemistry

Histology and immunohistochemistry procedures were performed as described [32] with minor modifications. Sections were incubated in 3% H_2_O_2_ for 15 min at room temperature, followed by boiling for 2 min and incubation for 20 min at 97°C in 10 mM sodium citrate (pH 6.0). Sections were incubated with 5% goat serum for 30 min, and subsequently with primary antibodies (ATRX D-19) at a dilution of 1∶50 overnight at 4°C. Secondary goat antibodies were used to recognize the primary antibodies. After washing, the horseradish peroxidase (HRP) conjugated polymer complex was visualized by incubation for 2 to 10 min with 3,3″-diaminobenzidine (DAB) substrate-chromogen. Sections were counterstained with methyl green, washed and mounted.

All images were taken at room temperature with a Retiga EX camera (Leeds Precision Instruments, Inc.) connected to a DMRA2 microscope (Leica). Image analysis was performed using Openlab 4.0.4 software (Improvision).

Growth plate morphology was analyzed by Safranin-O stain on P0.5 and P21 long bone sections. Sections were dewaxed, stained in hematoxylin followed by staining in fast green and safranin-O. Proportions of resting, proliferating and hypertrophic cells were determined using Openlab 4.0.4 software (Improvision) from at least three different mice.

#### Immunofluorescence of cultured primary chondrocytes

Primary chondrocytes were prepared from long bones of E15.5 mouse embryos[33]. Briefly, long bones were dissected, rinsed in PBS and incubated at 37°C for 20 min in trypsin-EDTA followed by digestion with 2 mg/ml collagenase P at 37°C for 2 h in Dulbecco's Modified Eagles Medium (DMEM) with 10% FBS. The cell suspension was filtered through a 70 μm cell strainer (Falcon), washed, counted and plated. For ATRX immunofluorescence, cells were grown on glass coverslips and fixed for 15 minutes in 4% paraformaldehyde, then blocked with 5% goat serum for 30 min at room temperature. Cells were then incubated with ATRX antibody at a 1∶300 dilution (H-300, Santa Cruz) and mouse anti-alpha tubulin at a 1∶10,000 dilution (Sigma) followed by FITC-conjugated anti-rabbit and Alexa 594-conjugated anti-mouse secondary antibodies. Slides were mounted in media containing DAPI and images were acquired on a Leica DMI 6000 b automated inverted microscope.

### Mouse breeding and genotyping

#### Ethics Statement

All procedures involving animals were approved by the University of Western Ontario Animal Care and Use Committee.

Mice were exposed to a 12-hour light–dark cycle and fed tap water and regular chow ad libitum. Mice conditionally deficient in ATRX were generated by crossing of *Atrx^LoxP^* females (129 sv background) [6] with heterozygous *Col2a1Cre*–knock-in male mice [22]. For developmental studies, midday of the day of vaginal plug discovery was considered E0.5. At scheduled times pregnant females were sacrificed by CO_2_.

PCR genotyping was performed from ear biopsy DNA for the presence of the Cre transgene as previously described [22]. Genotyping of embryonic and newborn mice was performed using PCR of DNA isolated from skin biopsies. PCR amplification was performed to detect the *Atrx* floxed alleles as previously described [8], the Cre transgene [22], as well as the sex determining region Y (*Sry*) gene to identify male mice. A 1.5 kb fragment of neo gene within the floxed allele of *Atrx* was identified with one set of primers (5′-GATCGGCCATTGAACAAGAT-3′ and 5′-ATA GGT CGG CGG TTC AT-3′) whereas the other set (5′-CCC GAG TAT CTG GAA GAC AG-3′ and 5′-ATA GGT CGG CGG TTC AT-3′) amplified a 600 bp fragment of wild type. Primers (5′-CCT GGA AAA TGC TTC TGT CC-3′) and (5′-CAG GGT GTT ATA AAC AAT CCC-3′) amplified a 300 bp fragment of the cre gene, whereas the other set (5′-GCA GGT GGA AAA GCC TTA CA-3′) and (5′-AAG CTT TGC TGG TTT TG GA -3′) amplified a 250 bp fragment of *Sry*. PCR conditions were as follows: 95°C for 3 min (95°C for 30 s, 55°C for 45 s, and 72°C for 1 min) × 36, 72°C for 10 min for *Cre* and *Sry*, 95°C for 3 min (95°C for 30 s, 55°C for 1 min, and 72°C for 5 min) × 36, 72°C for 10 min for *Atrx*.

### Skeletal stains and measurements

Live mice were weighed at P0, P7 and P21. Whole body length was measured using a ruler after sacrifice. For Alizarin Red/Alcian Blue staining, mouse carcasses were skinned and eviscerated, then fixed overnight in 95% ethanol followed by overnight fixation in acetone. Whole skeletons were placed in staining solution for 7–10 days (0.05% Alizarin Red, 0.015% Alcian Blue, 5% acetic acid in 70% ethanol)[32]. Skeletons were then cleared in 2% KOH. Images of stained bones were obtained with an Olympus SP-57OUZ. Limb bones and skulls from four different littermate pairs were measured using a dissecting microscope with a ruler.

### RT-PCR

Five micrograms of total RNA obtained from *Atrx^Col2cre^* and littermate control chondrocytes and control brains was reverse-transcribed using Omniscript RT kit (Qiagen) and used for PCR amplification with the following *Atrx*-specific primers: 17F (5′ –AGA ACC GTT AGT GCA GGT TCA-3′) for exon 17 and 20a (5′-ACC ACC ATC TTC TTG CCA TC -3′) for exon 20. Conditions for amplification were as follows: 95°C for 3 min (95°C for 30 s, 56°C for 30 s, and 72°C for 1 min) × 32, 72°C for 10 min.

### Western blot analysis

Primary rib cartilage was isolated from newborn mice. Ribs were dissociated using collagenase P and strained through a 70μm nylon filter to remove ossified tissue, followed by 3 days in culture over 1.5% agarose in PBS to specifically select for chondrocytes [34]. All tissues were lysed with MITO buffer (20 mM HEPES pH 7.5, 1 mM EDTA pH 8.0, 10 mM KCl, 1.5 mM MgCl_2_ and 1 protease inhibitor cocktail tablet [Complete mini, EDTA-free; Roche]), followed by treatment and lysis of nuclei with nuclear buffers without and with salt, respectively (20 mM HEPES pH 7.9, 450 mM NaCl (omitted for no salt) 1.5 mM MgCl_2_, 0.2 mM EDTA pH 8.0 and 1 protease inhibitor cocktail tablet). Nuclear buffer without salt was used for swelling nuclei, nuclear buffer with salt used for 25 minute nuclear lysis on ice. Extracts were quantified using the BCA protein assay (Sigma-Aldrich). Protein (5 μg) was resolved on a 6 % SDS-PAGE and transferred onto nitrocellulose membranes (Bio-Rad Laboratories). The membranes were probed with rabbit α-ATRX H300 (Santa Cruz Biotechnology, Inc.) followed by the appropriate horseradish peroxidase–conjugated secondary antibody (1∶5,000; GE Healthcare). After washing, the membrane was incubated in ECL before exposure using a ChemiImager 5500 (Alpha Innotech). The membrane was reprobed with mouse anti–beta-actin (1∶10,000; Sigma-Aldrich) as a loading control.
